# Simplex-Lattice Hydration Prediction and Microstructure Verification of Cementitious Systems

**DOI:** 10.3390/ma12030490

**Published:** 2019-02-05

**Authors:** Mohammad Iqbal Khan, Yassir M. Abbas, Galal Fares

**Affiliations:** Department of Civil Engineering, King Saud University, Riyadh 800-11421, Saudi Arabia; yabbas@ksu.edu.sa (Y.M.A.); galfares@ksu.edu.sa (G.F.)

**Keywords:** hydration, silica fume, PFA, thermo-gravimetric analysis, blended cement, simplex-lattice design

## Abstract

In this investigation, the age-dependent hydration development of blended pastes containing Portland cement (PC), pulverized fuel ash (PFA) and silica fume (SF) was assessed by quantifying the amount of CH and non-evaporable water using thermo-gravimetric analysis (TGA). Microstructure was investigated using scanning electron microscope (SEM). It was observed that the amount of liberated CH increases up to three-days in PC-PFA binary blended pastes, after which it progressively decreases and this reduction was proportional to the PFA dosage. The introduction of SF to PC-PFA binary mixtures to form ternary blended pastes has caused an early reduction of CH at one day where the majority of SF has been consumed during the first seven-days. The incorporation of 10% SF to PC-PFA pastes altered the low rate of hydration at early age. In addition, the presence of PFA showed insignificant influence on the non-evaporable water content until three-days then its effect became significant after seven-days. On the other hand, SF increased the non-evaporable water content from early ages up to seven-days. However, beyond 28 days, the presence of SF did not exhibit further pozzolanic activity. Furthermore, the ternary blended systems significantly increased the non-evaporable water content within three to seven days compared to the reference paste. Moreover, prediction nonlinear models of these hydration parameters were developed using the simplex-lattice design and validated against the experimental results. The latter have been further supported with SEM microstructural analysis showing good agreement between the predicted and realistic hydration.

## 1. Introduction

The addition of water to Portland cement (PC) leads to a series of chemical reactions known as cement hydration forming CSH (calcium-silicate-hydrates) and CH (calcium hydroxide), as the major hydration products. In these reactions, each of the silicates and aluminates phases undergoes a hydration process that ends up with the formation of its hydration product(s), which is also affected by the presence of other phases. The liberated CH crystals cause the fragility of the cementitious system as their resistance to crack propagation is negligible [1]. The pozzolanic materials, such as pulverized fly ash (PFA) and silica fume (SF), are industrial by-products with amorphous structures that have the potentiality to enhance the binder characteristics through their reactions with CH. The resultant improvement is attributed to their pozzolanicity that consumes the liberated CH, leading to the development of additional CSH of enhanced properties. Accordingly, their presence enhances the ability to develop strength through a densified microstructure [2,3]. Moreover, the application of PFA as a supplementary cementitious material (SCM) has been broadly implemented as it participates in sustainability, reduces greenhouse gas emission and improves workability at a low water-to-binder ratio (w/b). However, the slow rate of strength improvement over a short-term represents the main drawback of PFA application in concrete. On the other hand, at later-ages, the entire strength and durability could be improved with respect to the control mixture. To overcome this early hydration issue, the introduction of SF becomes a feasible solution imparted by its high early pozzolanic activity.

Thomas et al. [4] reviewed the reported researches related to modelling and simulating the complex hydration and microstructure of cement-based materials. These models included the single particle, mathematical growth and lattice methodologies. Moreover, the modern methods of molecular-scale modelling are capable of simulating the development of CSH phase and modelling the hydration kinetics. It was concluded that the future development of a comprehensive understanding of the hydration process is highly associated with these simulations and models. The simplex-lattice method offers an opportunity to predict the hydration properties of cement-based blended systems [5]. This technique is a systematic arrangement of consistently separated data-points on a mesh (lattice) used to develop the appropriate response surface (regression equations). This method differs primarily from the conventional regression technique in their unique stipulations of the observed data-points within the prospective simplex lattice. This lattice is typically designated as [*q*, *m*] which reveals that the associated regression equations is of m-order for a mixture of *q*-components. The entire coefficients of these regression equations describe the influence of mixture components on the response surface. It is worth mentioning that in the 50th of the preceding century, the statistical research body established the theory of the simplex-lattice design method and its initial applications [5]. Claringbold [6] was the first researcher, who reports the simplex-optimization of a three-ingredient blend. Later, Scheffé [7] has launched the idea of simplex-lattice regression system. In the area of civil engineering, Chen et al. [8] have implemented the simplex-lattice design method to establish linear and cubic prediction equations of the strength of multi-cementitious-component concrete. Recently, Abbas [9] has utilized this method to design the concrete with SCMs with respect to its strength and permeability properties. Despite the rational degree of success that the simplex-lattice design method has achieved in the optimization of multi-component mixtures, there is no available report on the approachable literature on its use for the prediction of hydration properties of blended cement materials.

The development of the hydration of cementitious materials is usually assessed through the determination of CH content, which is commonly quantified by: (i) thermo-gravimetric analysis (TGA), (ii) X-ray diffraction (XRD) and (iii) monitoring heat release using isothermal calorimetry (ISC) [2,10,11,12,13,14,15,16]. These methods are based on different measurement principles for the CH content that presents in the hydrating paste, which causes a wide variation in the results [17]. Midgley [12] compared ISC, XRD and TGA methods for measuring CH content in cement paste and concluded that the TGA is the most reliable technique, which provides the smallest errors of ±0.05%. It is worth noting that TGA is a method by which the temperature-dependent weight of a material is measured at a uniform rate of raising temperature. Other techniques of analysis for CH content are available in the literature, such as scanning electron microscopy (SEM), non-contact impedance measurement (NCIM) and pore solution analysis [18,19]. Moreover, Deboucha et al. [20] have presented a modified TGA method by approximating the dosage of SCM that increases the volume of the hydration products. In this investigation, the age-dependent development hydration of blended pastes containing PC, PFA and SF was assessed, where the amount of CH and non-evaporable water were quantified using TGA. Moreover, prediction models of these hydration parameters were developed using the simplex-lattice design and validated against the experimental results. Eventually, the validated nonlinear models were utilized to investigate the influence of PFA and SF on the hydration parameters in their ternary cement systems of PC-PFA-SF. SEM technique is supportive of the hydration and modelling. It has been frequently used in several studies concerned with characterization, mixture design and modelling [21,22,23]. Accordingly, the current approach has been supported with SEM microstructural analysis to provide the current approach a realistic visualization and imaging of the hydration process.

## 2. Materials and Methods

### 2.1. Materials, Mixing and Casting

**Figure 1:**
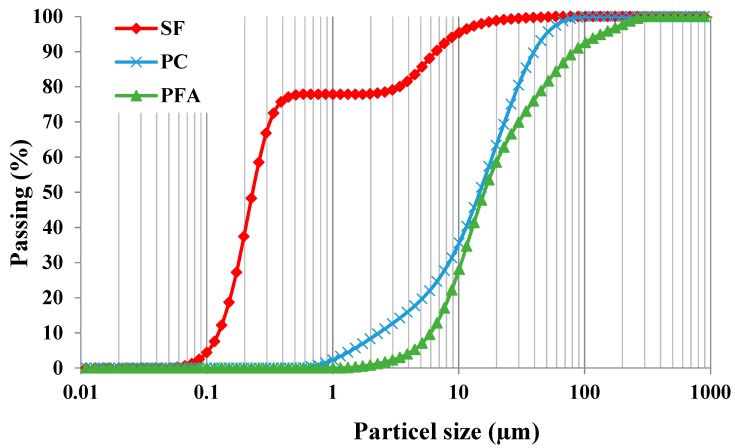
Particle-size distribution analysis of fine powders.

**Figure 2:**
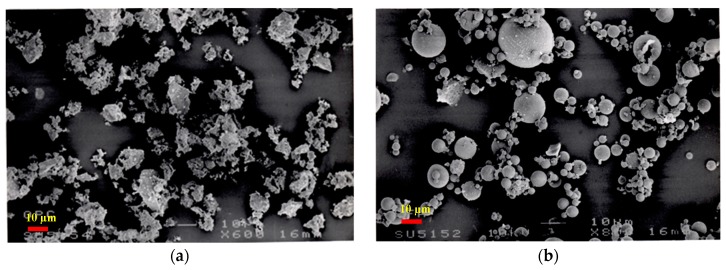

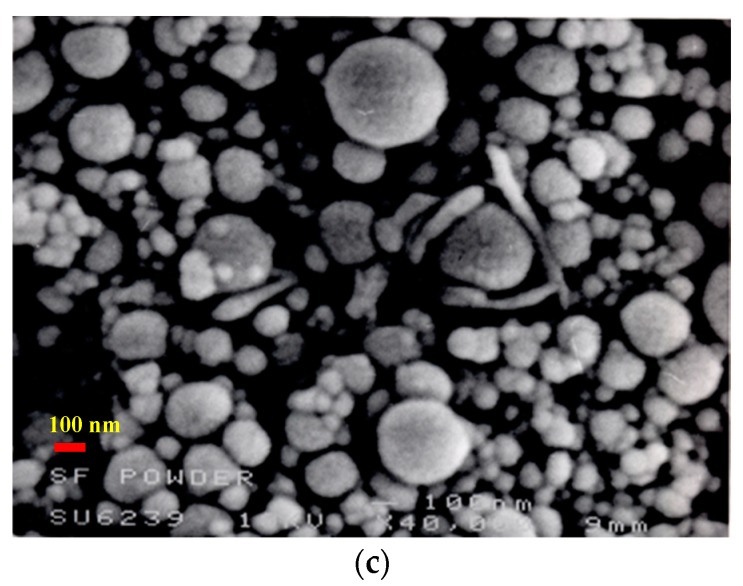
Back-scattered electron detector images of fine powders. (**a**) PC; (**b**) PFA; (**c**) SF.

In this investigation, PC and PFA conforming to BS EN 197-1:2011 and BS EN 450-1:2012, respectively, have been employed. Additionally, the utilized SF was in the form of a semi-liquid mixture with 50% moisture content. PC was used as the main binder, which complying with ASTM C150 specifications and that has been partially replaced by PFA and SF on mass basis. The particle-size distribution analysis of PC, PFA and SF is shown in Figure 1. PC and PFA had median particle sizes of 15 and 16 µm, respectively, while the median particle size of SF was about 200 nm. The use of SEM analysis confirmed the median particle size obtained for PC, PFA and SF, as demonstrated in Figure 2. It was also confirmed that the particle sizes of SF were in the range of 50 to 400 nm. Furthermore, a super-plasticizer (SP) of sulfonated naphthalene formaldehyde in condensate aqueous solution was exploited to adjust the flowability of the mixtures in the range 200 ± 10 mm. Table 1 shows the components of the cementitious materials (PC, PFA and SF) for the 16 developed mixtures. It should be noted that for these mixtures, the w/b ratio was kept constant at about 0.3. Moreover, the water contents of SF and SP were taken into consideration to correct for the mixing water content.

Mixing was performed using electric hand blender (Hobart, OH, USA). To inhibit water drying-up, the mixtures were cast in plastic cups, carefully consolidated and airtight. It is worth noting that the paste specimens were reserved indoor and demolded one day after casting and retained in water tanks (at a temperature of 20 ± 3 °C) to the intended age of testing (1, 3, 7, 28, 90 and 180 days). Then, the samples were taken out of the mist room and placed in an oven set at 105 °C. The samples were kept in an oven for drying until constant weight was achieved, usually after 12 h. Later, the samples were ground to fine powder that passed through a 75 μm sieve in order to improve the uniformity of the sample. The process of grinding was conducted as quickly as possible (usually within 5 min) to minimize the carbonation. The powdered samples were sealed in airtight small glass vials, which immediately loaded in the thermo-balance for testing.

### 2.2. Experimental Methods

At a temperature level between 420 °C to 550 °C, the dehydroxylation of CH eventuates, as presented in Equation (1). This equation reveals that the dehydroxylation of one molar mass of CH (74 g/mol) releases one molar mass of water (18 g/mol). Moreover, Equation (2) represents the chemical reaction when carbonation occurs. Accordingly, the measurement of CH as TGA result is rectified using the weight of CaCO_3_ (Calcium carbonate). As the temperature increases from 550 °C up to 780 °C, the CO_2_ (carbon dioxide) evolves, hence, decarbonization is being the consequence (Equation (3)). The Equations (2) and (3) demonstrate that the carbonation of CH forms one gram-molecular weight of CaCO_3_, which in turn releases one molar mass of CO_2_ through decarbonization reaction. Thus, the reduction in the weight equivalent to releasing CO_2_ corresponding to carbonation of CH is primarily existed in the cement paste.

Ca(OH)_2_ ⟹ CaO + H_2_O (dehydroxylation) (74 g)            (18 g)(1)

Ca(OH)_2_ + CO_2_ ⟹ CaCO_3_ + H_2_O (74 g)          (100 g)(2)

CaCO_3_ ⟹ CaO + CO_2_↑ (Decarbonization) (100 g)            (44 g)(3)

In this investigation, care was taken to prevent the carbonation during sample preparation and the test was carried out in a nitrogen environment. Despite this careful consideration, the probability of CH carbonation exists permanently [24]. The weight loss above 550 °C is mainly due to CO_2_ release. However, weight loss above 550 °C can also be caused due to the final stages of dehydration of CSH and hydrated aluminate phases, which may not be distinguished from TGA evidence alone [25]. Considering the persistent probability of carbonation, the measurement of CH requires an information concerning the decrease in the weight of the paste at a temperature beyond 550 °C. For this reason, the total weight of CH can be measured through within the temperature ranges of 420 °C to 550 °C and above 550 °C). The total amount of CH was estimated using Equation (4), in which, *W*_CH_ is the total amount of CH (%), while *W*_2_ refers to total weight loss between 420 °C to 550 °C and *W*_3_ denotes the weight loss between 550 °C and above.

(4) $$W_{\mathrm{CH}}=\frac{74}{18}W_{2}+\frac{74}{44}W_{3}$$

On-evaporable water is that involved in CH and other calcium-based products of the cement hydration. This water is usually used to represent the degree of hydration of plain PC. However, the amount of this water may not be a clear-cut clue of the hydration level when PC is blended with other cementitious materials. This ambiguity is attributed to: (i) the exact content and the stoichiometry of the hydration outcomes and (ii) the delicate differentiation between the evaporable water and the coupled one due to chemical bond is obscure. For instance, a noticeable weight loss of hydrate water from calcium-sulpho-aluminates and tri-calcium-aluminate-hydrates is experienced at 105 °C [11]. Unlike PC hydration products, the chemical reactions of pastes with pozzolanic materials consume CH with an amount of water to develop hydration products of unconventional composition. Therefore, the amount of non-evaporable water is the sole utility for comparison purposes. Additionally, the amount of “hydrated water” is that related to the amorphous silica-alumina hydration products. This amount is evaluated by deducting the water content of CH from that of the non-hydrated water. However, hydrated water content theory may not be applicable in the systems where some weight loss is observed during the decarbonization process. It is worth noting here that Marsh and Day [26] have reported negligible weight loss during the decarbonization reaction process.

**Figure 3:**
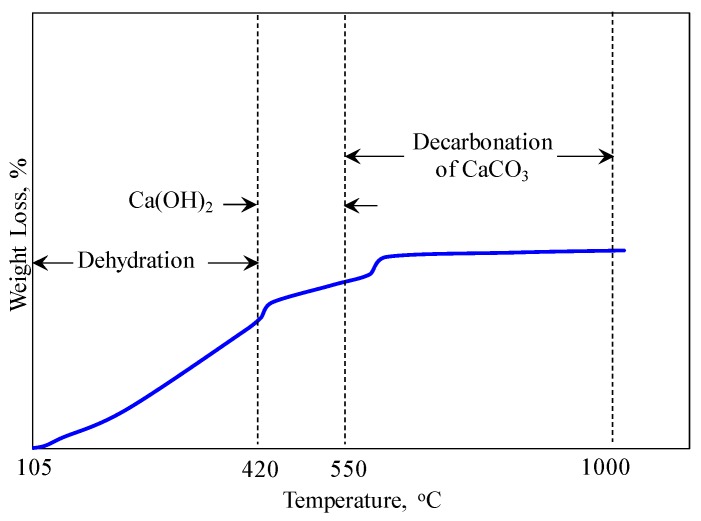
Typical thermogravimetric analysis (TGA) thermogram showing weight loss vs. temperature curve of cement pastes.

In this investigation; however, it was assumed that some carbonation is taking place. For blended systems, a more realistic approach is to determine the non-evaporable water content by using the weight loss associated with dehydration reactions only. This process is related to the weight loss between temperatures 105 °C to 400 °C and takes into account the amount of combined water associated with the calcium silicates. In the past, some researches selected a temperature up to 400 °C to evaluate the amount of combined water of calcium silicates only [27]. Therefore, in this investigation, the non-evaporable water content was determined in the range of temperature of 105 °C and 400 °C. The equipment used in this investigation was thermogravimetry balance Stanton Redcroft model TG-760 (Stanton Redcroft Limited, London, UK). The instrument consists mainly of an electronic microbalance, a furnace and an operational programmer unit. The sample size used was 20–24 mg and the test was performed under nitrogen gas flow rate of 15 mL/min. The samples were heated between the temperatures of 105 °C to 1000 °C. The clarity of these peaks depends on the degree of heating and different modes adopted for the test. Therefore, for the clarity of peaks, a slow rate of heating was employed (10 °C/min). The furnace was cooled by circulating water in order to achieve a fast cooling rate. The thermos-balance was connected to the plotter at the chart speed of 12 cm/h and the raw data curves were obtained. These curves were analysed for the weight loss of sample at various stages by following a method proposed by El-Jazairi and Illston [28,29]. Figure 3 shows the typical relation of the paste weight loss with respect to temperature change in the range of 105 °C to 1000 °C. The weight loss in this relation may be divided into (i) 105 °C to 420 °C (the dehydration reactions take place to CSH phases and other products), (ii) 420 °C to 550 °C (de-hydroxylation of CH exists) and (iii) 550 °C to 780 °C (decarbonation of the CaCO_3_). Additionally, microstructural analysis of paste mixture was conducted using electron backscattering diffraction technique (EBSD) of analysis, model Jeol JSM-6610LV (Geol, Tokyo, Japan) coupled with an Oxford INCAx-ACT X-ray microanalyzer (Oxford instruments, Oxfordshire, UK).

### 2.3. Simplex Lattice Design

**Figure 4:**
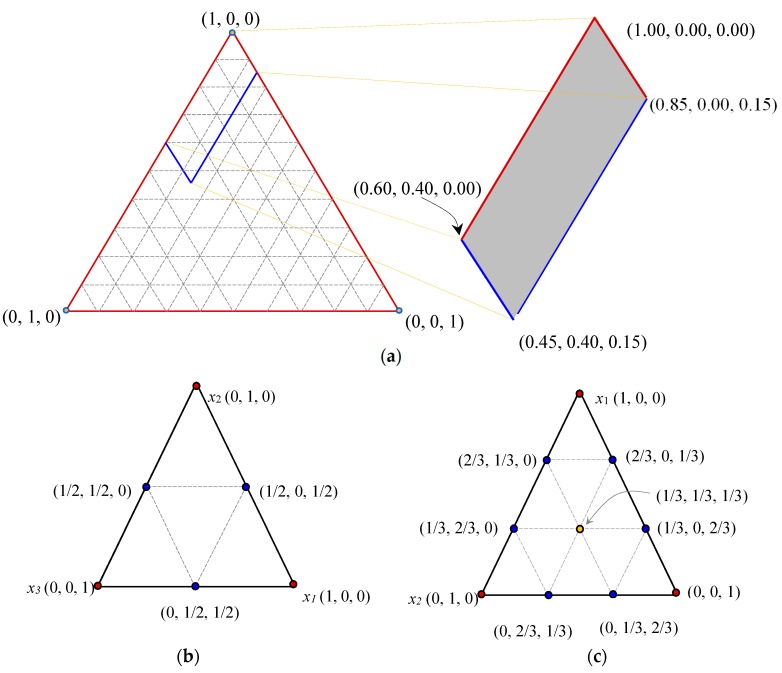
The simplex-lattice equilateral triangular and the corresponding pseudo-coordinates of a three-component mixture: (**a**) Entire lattice, (**b**) [3, 2] lattice and (**c**) [3, 3] lattice.

For a mixture with [*q*, *m*] simplex-lattice, hypothesizing that *q* is the number of components and *x_i_* are the pseudo-ingredients (dosages) of the *i^th^* component in the lattice. As indicated by Equation (5), *x_i_* should satisfy zero-one bounds and sum-to-zero conditions. Simplex-lattice “normalized” coordinates systems is frequently addressed as (*x*_1_, *x*_2_, …, *x_q_*) with partial or full lower and/or upper component limits. Equation (6) presents the postulated ingredient limits of *x_i_*, in which, *x*_1_, *x*_2_ and *x*_3_ denote the proportions of PC, PFA and SF, respectively. Additionally, Figure 4 shows the analogous investigational region with its extremity peaks and the corresponding quadratic and cubic regression model’s coordinates of a three-ingredient blend. Moreover, Equation (7) reveals that *x_i_* have (*m* + 1) rooms in the corresponding limits (zero-to-one) of the [*q*, *m*] simplex-lattice. Where, the total number of the (*n*) of the [*q*, *m*] data-points could be evaluated from Equation (8). Following the Scheffé [7] scheme, the actual coordinates {*z*} were transformed to the pseudo-components {*x*} using Equation (9) through the transformation matrix [*T*].

(5) $$0\leq x_{i}\leq 1\;\mathrm{and}\;\overset{q}{\underset{i=1}{\sum }}x_{i}=1;\;i=1,\;2,\;\ldots ,\;q$$

(6) $$0.45\leq x_{1}\leq 1;\;0\leq x_{2}\leq 0.4;\;0\leq x_{3}\leq 0.15$$

(7) $$x_{i}=0,\frac{1}{m},\frac{2}{m},\;\ldots ,\;1$$

(8) $$n=\frac{q\left(q+1\right)\left(q+2\right)\ldots \left(q+m-1\right)}{m\left(m-1\right)\left(m-2\right)\ldots .\left(1\right)}$$

(9) {z}=[T]{x}

Moreover, Equation (10) displays the commonly used simplex-lattice regression model, which fits the observed data-points of the [*q*, *m*] simplex-lattice. Equation (10) has *n*-terms (Equation (8)), starts by a constant term and ends with that of *m*-order. It should be noted that Equation (11) is exclusively coherent if the limits of Equation (5) are incorporated, which implies that the constants of Equation (10) (*α_i_*, *α_ij_*, *α_ijk_*, …) are autonomous. Plugging Equation (11) in Equation (10) phases out the autonomy of *x_i_* terms, which alters the regression model to be of (*q* − 1) ingredients (i.e., with (*n* – 1) coefficients); however, the order of the function remains the same (of *m*-order). This alteration of the regression equations ignores the information of the *q*-ingredient and its associated impacts. Avoiding the ambiguity of the *q*-ingredient, a different method was utilized, which requires reproducing Equation (10) by incorporating the sum-to-zero conditions (Equation (5)) into some of its terms and streamlining the produced equation. This form of the polynomial is commonly known as “canonical” model with *n* coefficients, which equals to the data-points of the [*q*, *m*] simplex-lattice. Accordingly, Equations (12) and (13) give the quadratic and cubic “canonical” models, respectively. Evidently, six and ten observed results must be retrieved to develop the quadratic and cubic “canonical” models, respectively. This involve the solution of a group of simultaneous equations with the terms of the pseudo-coordinates as variables and the observed result as the resulting answer.

(10) $$f_{\left(x_{i},x_{j},x_{k},\ldots \right)}^{\left(m\right)}=\alpha_{o}+\overset{q}{\underset{i=1}{\sum }}\alpha_{i}x_{i}+\overset{q}{\underset{1\leq i<j\leq q}{\sum }}\alpha_{ij}x_{i}x_{j}+\overset{q}{\underset{1\leq i<j<k\leq q}{\sum }}\alpha_{ijk}x_{i}x_{j}x_{k}+\ldots$$

(11) $$\alpha_{o}=\delta_{o}x_{i};\;x_{i}^{2}=x_{i}\left[1-\sum_{\begin{array}{c} j=1\\ j\neq i \end{array}}^{q}x_{i}\right];\;x_{i}^{3}=x_{i}^{2}\left[1-\sum_{\begin{array}{c} j=1\\ j\neq i \end{array}}^{q}x_{i}\right];\;\ldots$$

(12) $$f_{\left(x_{1},x_{2},x_{3}\right)}^{\left(2\right)}=\beta_{1}x_{1}+\beta_{2}x_{2}+\beta_{3}x_{3}+\beta_{12}^{\left(2\right)}x_{1}x_{2}+\beta_{13}^{\left(2\right)}x_{1}x_{3}+\beta_{23}^{\left(2\right)}x_{2}x_{3}$$

(13) $$f_{\left(x_{1},x_{2},x_{3}\right)}^{\left(3\right)}=\beta_{1}x_{1}+\beta_{2}x_{2}+\beta_{3}x_{3}+\beta_{12}^{\left(3\right)}x_{1}x_{2}+\beta_{13}^{\left(3\right)}x_{1}x_{3}+\beta_{23}^{\left(3\right)}x_{2}x_{3}+\mu_{12}^{\left(3\right)}x_{1}x_{2}\left(x_{1}-x_{2}\right)\;+\mu_{13}^{\left(3\right)}x_{1}x_{3}\left(x_{1}-x_{3}\right)+\mu_{23}^{\left(3\right)}x_{2}x_{3}\left(x_{2}-x_{3}\right)+\beta_{123}^{\left(3\right)}x_{1}x_{2}x_{3}$$

## 3. Results and Discussion

Table 2 and Table 3 present the percentages of CH and non-evaporable water of the investigated binary and ternary cement pastes at various ages, respectively.

### 3.1. CH Content

#### 3.1.1. Effect of PFA on CH Content

**Figure 5:**
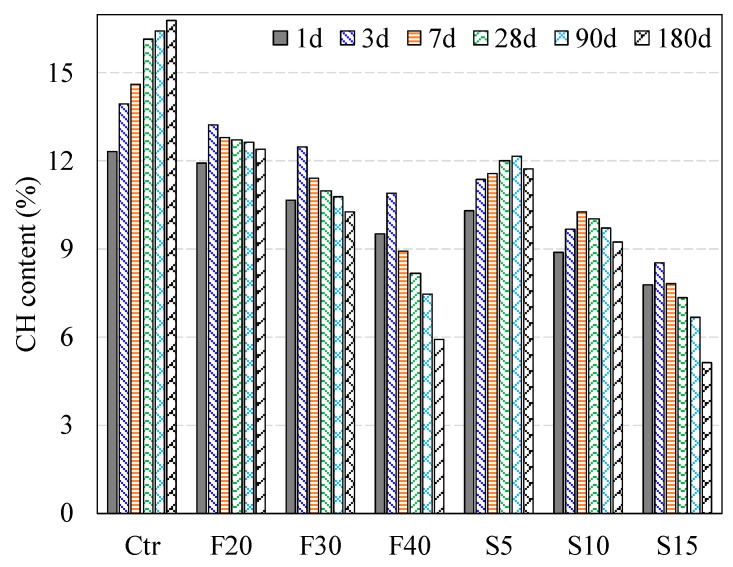
CH content in PC control and binary blended paste systems with w/b of 0.3, mist cured, at various ages.

**Figure 6:**
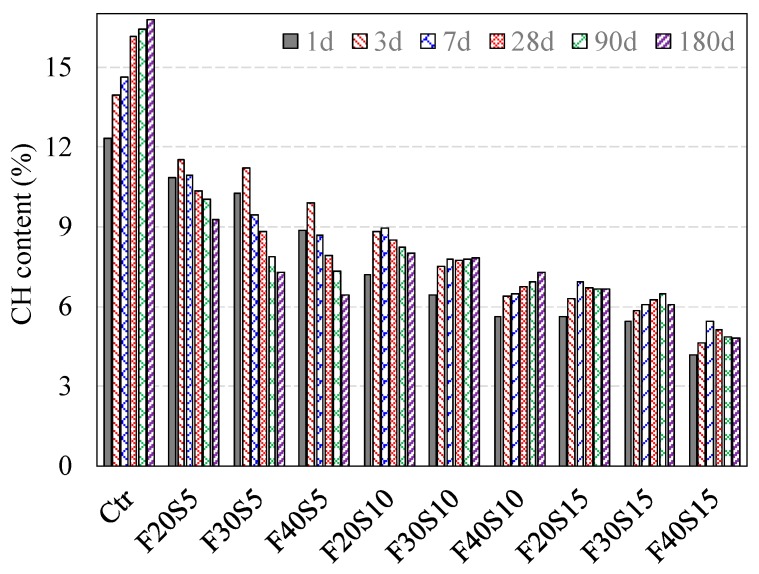
CH content in PC control and ternary blended paste systems with w/b ratio 0.3, mist cured, at various ages.

Table 2 presents the percentages of CH of the investigated binary and ternary cement pastes at various ages, respectively. Moreover, Figure 5 demonstrates the CH content in binary systems containing PFA and SF as cement replacements with w/b ratio of 0.3 at various investigated ages. Moreover, the CH content in ternary systems at the ages between 1 and 180 days is shown as in Figure 6. Figure 5 demonstrates that the amount of CH decreases as the dosage of PFA increases. Moreover, the mixes with 20%, 30% and 40% PFA revealed the CH contents of 11.9%, 10.7% and 9.5%, respectively at one-day of hydration with respect to 12.3% CH content of pure PC paste. In the regard of hydration time, the amount of CH of pastes containing PFA rises- for short-term curing (up to three-days), however, this amount falls-off steadily after three-days of mixing. It is worth noting that these findings concur with that reported by Weng et al. [30]. After 1-day, the reduction of CH content for the pastes containing PFA concerning the reference paste reveals that the introduction of PFA consumes CH, which attributed to the lower PC content. The reduction of CH amount after three-days brings to mind the initiation of pozzolanic reaction [30]. Furthermore, the incorporation of PFA lowers the production of CH for immature pastes (with up to three-days age). This finding is in agreement with these of Fajun et al. [31].

#### 3.1.2. Effect of SF on CH Content

The paste containing 5% SF exhibited 10.3% and 11.4% CH at 24 h and three-days, respectively (Figure 5). At early age (up to three-days), the production of CH is preponderance, however, after that insignificant increase was observed. This CH slow-down production is associated with lacking additional SF to promote its consumption throughout the hydration reaction. For this reason, it could be expected that most of the 5% SF was employed during the first three-days. This finding is in agreement with Yogendran et al. [32] as they stated that 5% SF mostly reacts in the early two-days. The paste with 10% SF demonstrated a similar pattern to that of a paste containing 5% SF up to the age of seven-days but exhibited lower CH content than that mixture with 5% SF. Following seven-days, it decreases gradually until reaching 9.2% at 180 days, which is about 7.5% lesser than that of the plain PC mixture. Similarly, the mix containing 15% SF showed 7.8% CH at 24 h, while at three-days it increases slightly. Beyond the three-days, a gradual decrease was registered, reaching 5.1% at 180 days (about 11.7% lower than that of plain PC mix).

It is observed from the above results that the introduction of SF notably reduces the amount of CH after the initiation of the hydration reaction by 24 h. Cheng and Feldman [33] reported similar results where a reduction in CH begun at eight hours for SF mixtures with w/b ratio of 0.60. This reduction may arise earlier than one-day and could be referred to as a couple of unconnected influences. The first influence corresponds to the elimination of CH from the paste containing SF that is also indicated in the results of Li et al. [34]. However, the second influence is related to the accelerated pozzolanic reaction between SF and Ca^+2^ (calcium cations). For w/b of 0.3, the SF extends the inactive hydrations cycle and retards the rise-up of active reaction over entire temperature [30]. Cheng and Feldman [33] also reported similar findings where SF paste decreased the amount of CH at the age of one-day for various w/b ratios (0.25, 0.45 and 0.60). In their investigation, this change of CH content was referred to as the consequence of the reaction of Ca^+2^ and SF after about eight hours of mixing. Moreover, Grutzeck et al. [35] reported that Ca^+2^ might produce an additional phase when physically taken in the top of the SF. This finding reveals that the premature reaction of SF and Ca^+2^ in the blended paste is achievable as established by Larbi et al. [36]. These investigators observed a dramatic drop in the concentration of Ca^+2^ in paste containing SF within four hours. This decrease demonstrates the development of CSH because of the accelerated pozzolanic reaction at short-term (up to three-days) between Ca^+2^ and SF that was absorbed in the paste with a notable amount.

#### 3.1.3. Simplex Lattice Prediction Models and Response Surface Contours

Assuming that Equations (12) and (13) give the quadratic and cubic forms of the CH content response (*f_CH_*) as a function of the pseudo-coordinates *x*_1_, *x*_2_ and *x*_3_ (components of PC, PFA and SF, respectively). The regression parameters of these nonlinear models (Equations (14) and (15)) were evaluated by correlating the actual- to pseudo-coordinates with the relevant observed CH content (Table 4). It should be noted that the developed quadratic and cubic prediction equations were based on the 28-day CH content (Table 2). The following findings were made from Equations (14) and (15). As it is the main producer of CH, we noticed that the influence of PC for mono-ingredient pastes on the CH content is notably more than those of PFA and SF (16.17 > 8.10 > 7.35). However, depending on the replacement level, the CH content of the paste containing only PFA is comparable to that with merely SF (8.10 ≈ 7.35). Additionally, Equation (14) (the quadratic model) illustrates that the PC-PFA and PC-SF binary systems have a positive impact on the release of CH, however, the PFA-SF binary blend has a negative influence. Contrarily, the third order model (Equation (15)) shows that PC-SF binary system has a negative impact on the CH content, however, the PFA-SF binary blend has a positive effect. However, Equations (14) and (15) have an agreement with regard to the influence of the binary combination of PC and PFA. Moreover, the third order models of the CH content (Equation (15)), suggest that the ternary blend of PC-PFA-SF has the “synergistic” consequence on the release of CH of the paste.

**Figure 7:**
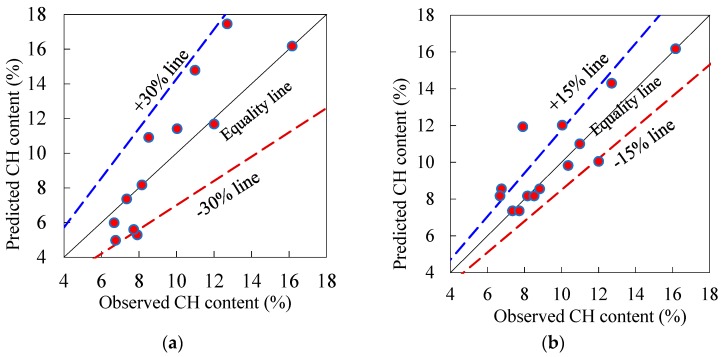
Actual vs. predicted 28-day CH content (%) of control and ternary blended pastes: (**a**) Quadratic model (Equation (14)) and (**b**) Cubic model (Equation (15)).

Additionally, Figure 7 related the observed and predicted (using the quadratic and cubic models of Equations (12) and (13)) 28-day CH content of the 14 pastes. This figure noticeably reveals that the majority of the predicted results are close to the analogy (predicted = observed) line with the mean absolute inaccuracy of 1% and 11% for the quadratic and cubic models (Table 4), respectively. It is worth noting that the observed-predicted points under the analogy line show an underestimation in the CH content, whereas, those points above the equivalence line demonstrate an overestimation in the prediction. Figure 7 indicates that both the quadratic and cubic models exhibit rational prediction behaviour, as the distribution of the data points is virtually even around the equality line. Additionally, the error band of the quadratic and cubic models are ±30% and ±15%, respectively. As the suggested cubic model of the CH content exhibits entirely superior prediction performance (with a narrow banded error), it will be employed with a practical precision for the estimation of the CH content of multi-component pastes of PC-PFA-SF in the next sections.

(14) $$f_{CH}{}^{\left(2\right)}=16.17x_{1}+8.16x_{2}+7.35x_{3}+2.22x_{1}x_{2}+17.02x_{1}x_{3}-11.76x_{2}x_{3}$$

(15) $$f_{CH}{}^{\left(3\right)}=16.17x_{1}+8.16x_{2}+7.35x_{3}+2.159x_{1}x_{2}-11.6x_{1}x_{3}+14.73x_{2}x_{3}+4.2x_{1}x_{2}\left(x_{1}-x_{2}\right)\;-28.446x_{1}x_{3}\left(x_{1}-x_{3}\right)-15.135x_{2}x_{3}\left(x_{2}-x_{3}\right)+21.071x_{1}x_{2}x_{3}$$

#### 3.1.4. Effect of the Combined PFA and SF on CH Content

**Figure 8:**
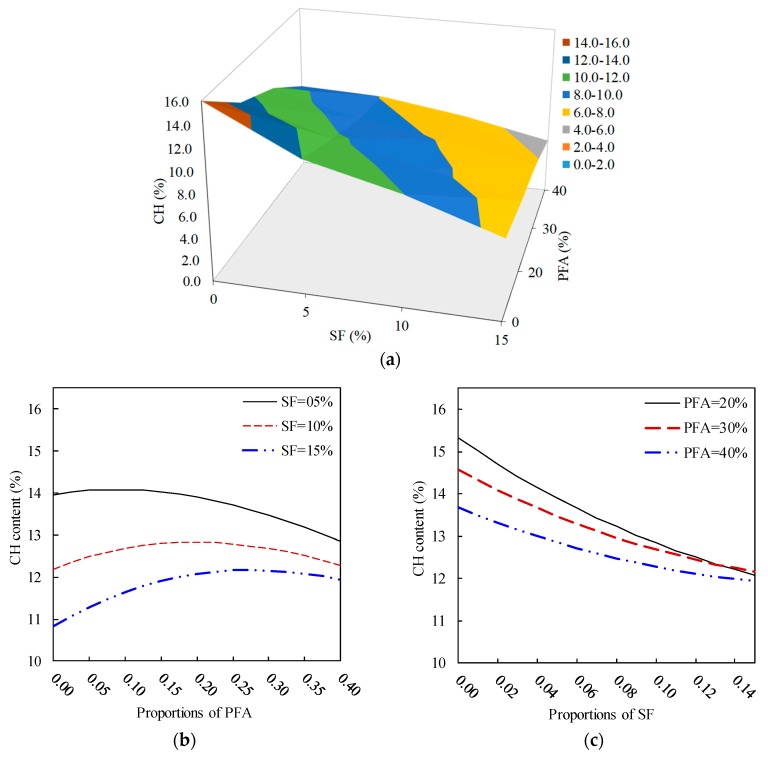
Effect of the PFA and SF on the CH content of ternary blended systems. (**a**) response surface, (**b**) constant SF, (**c**) constant PFA.

Figure 8 shows the coupled influence of introducing PFA and SF to a cement mixture on its CH content. Figure 8a establishes the three-dimensional combined effect of PFA and SF on CH content based on the observed findings, which is presented here for comparison purpose. Figure 8b demonstrates that CH content increases as the PFA content increases, which indicates that the PFA has its own CH content. This behaviour is also observed experimentally (Figure 8a), which confirms the capability of the prediction model. Moreover, the amount of the CH content was proportional to the dosage of SF (the growth was 4.5%, 5.2% and 12.4% for 5% SF, 10% SF and 15% SF, respectively). However, the entire modification was insignificant. Additionally, Figure 8c illustrates that the CH content decreases with increasing the SF content for a constant amount of PFA and a higher CH content for the lower PFA dosage. However, at SF more than 8%, the PFA content becomes of no significant influence on the CH content of the blended paste.

From Figure 8b, the effect of PFA content on the CH presence is significant at lower PFA content, whereas, its narrows down with an increased amount of PFA (up to 40%). The presence of SF caused a reduction in CH content, however, it tends to converge with presence of PFA content of 40%. For instance, at 5% SF + 0% PFA and 15% SF + 0% PFA the CH contents are 14% and 10.8%, respectively whereas at by adding 40% PFA to these mixes the CH contents converge to about 13% and 11%, respectively. Similar trends are shown in Figure 8c, which shows convergence of CH content of all mixes containing SF and PFA. Clearly, there is great influence of SF and PFA in reducing the 28-day CH content, however, it is limited to the amount of SF and PFA and optimum range of SF and PFA, which was close to 8–10% and 25–30%, respectively.

### 3.2. Non-Evaporable Water Content

**Figure 9:**
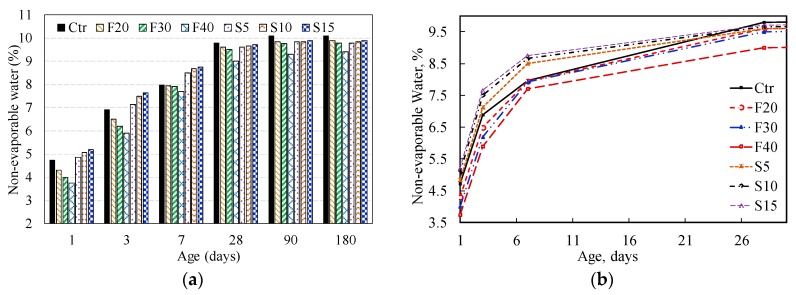
Non-dehydrated water content in control and binary blended paste systems with w/b ratio 0.3, mist cured, at ages (**a**) 1, 3, 7, 28, 90 and 180 days (**b**) 1, 3, 7 and 28 days.

**Figure 10:**
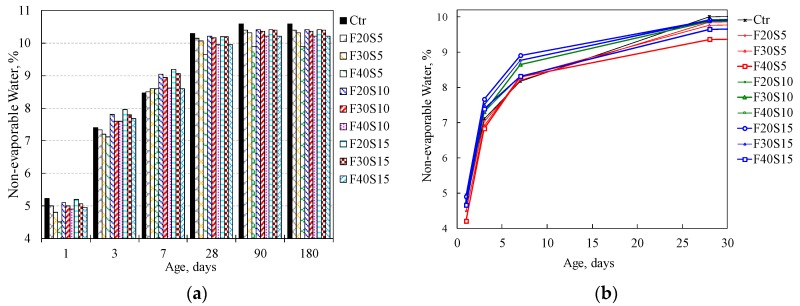
Non-dehydrated water content in control and ternary blended paste systems with w/b ratio 0.3, mist cured, at ages (**a**) 1, 3, 7, 28, 90 and 180 days (**b**) 1, 3, 7 and 28 days.

Table 3 shows the content of the non-evaporable water of the investigated binary and ternary cement pastes at various ages, respectively. In this investigation, the non-dehydrated water content was determined using Equation (5). The non-dehydrated water content of the control and binary blended systems incorporating PFA and SF is exhibited as shown in Figure 9. While, the non-evaporable water content in ternary systems of paste can be seen in Figure 10. The plain PC paste showed a continuous increase in non-evaporable water content up to 28 days, the rate of increase is retarded with an increase in age while a slight increase was observed beyond 28 days.

#### 3.2.1. Influence of PFA on Non-Evaporable Water Content

Figure 9 demonstrates that for short-term hydration (up to three-days) the amount of non-evaporable water decreases as the dosage of PFA increases. This reduction suggests that at early ages, a lower content of PC is dedicated for the hydration reaction and PFA seems to be inactive as latent pozzolanic reaction turns up. At seven days, the non-evaporable water content of PFA pastes was approximately the same as that of plain PC paste, indicating the creation of accelerated hydration by PFA. After 28 days, these PFA paste mixes attained lower values, which however increases slowly.

#### 3.2.2. Influence of SF on Non-Evaporable Water Content

Figure 9 shows that up to seven-days the pastes with SF had high non-evaporable water content with respect to the reference (with plain PC) paste. This increase is associated with the production of extra CSH gel because of the accelerated pozzolanic reaction between SF and CH. However, SF pastes showed slightly lower non-evaporable water content after 28 days when compared to the reference paste. Further, at early ages (up to seven-days) the non-evaporable water content increased with an increase in SF content; however, at 28 days and beyond there is insignificant variation.

#### 3.2.3. Simplex Lattice Prediction Models and Response Surface Contours

Equations (16) and (17) are the quadratic and cubic prediction models, for the 28-day non-dehydrated water content (*f_W_*). The constants of these equations were evaluated by substituting the pseudo-coordinates and the pertinent non-evaporable water content those presented in Table 4. With respect of the behaviour of the developed nonlinear models for the 28-day non-dehydrated water content (Equations (16) and (17)), we noticed that for a single-component paste, the three cement components (PC, PFA and SF) have an equivalent effect on $f_{W}$ (9.8 ≈9≈9.7). Unlike $f_{CH}^{\left(2\right)}$, $f_{W}^{\left(2\right)}$ (Equation (16)) suggests that the binary blends of PC-SF and PC-SF are incompatible. This incompatibility is attributed to their negative impact on increasing the non-evaporable water content. Additionally, the pair combination of PC-PFA is synergistic for rising the non-evaporable water as well as the CH contents. Typical influences for the binary systems can be drawn based on the cubic model of the non-evaporable water content (Equation (17)). Additional significant finding base on Equation (17) is that the ternary combination of PC-PFA-SF has the “antagonistic” result on the discharge of non-evaporable water of the paste.

**Figure 11:**
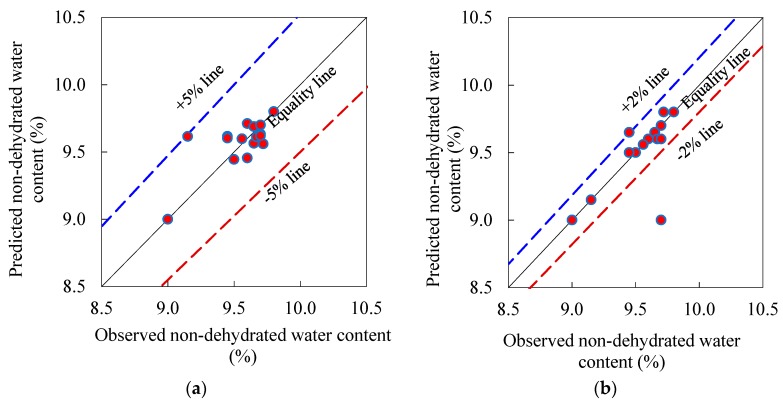
Actual vs. predicted 28-day non-dehydrated water content (%) of control and ternary blended pastes: (**a**) Quadratic model (Equation (16)) and (**b**) Cubic model (Equation (17)).

Moreover, the interrelation between the observed and predicted (based on the quadratic and cubic models (Equations (16) and (17)) non-evaporable water content are presented in Figure 11. Noticeably, this figure indicates that significant number of data points are adjacent to the equality line. In addition, Table 4 shows that the entire average error of the quadratic and cubic models predictions were 0% and 2%, respectively. Concerning the distribution around the equality line and the non-broader band error (±5% and ±2%), Figure 11 illustrates that the nonlinear prediction models of the non-evaporable water content have reasonable performance. The enhanced accuracy of the prediction models of the non-evaporable water content with respect to those for the CH content is attributed to the natural notably tiny range of the non-evaporable water content observed results (only 0.8 range, Table 4). In the next sections, the cubic prediction model of the non-evaporable water content will be used to investigate the influence of the various parameters, as it shows better prediction capability (only ± 2% error band, Figure 11) with respect the quadratic model.

(16) $$f_{W}^{\left(2\right)}=9.8x_{1}+9x_{2}+9.7x_{3}+0.8x_{1}x_{2}-0.76x_{1}x_{3}+x_{2}x_{3}$$

(17) $$f_{W}^{\left(3\right)}=9.8x_{1}+9x_{2}+9.7x_{3}+0.676x_{1}x_{2}-0.653x_{1}x_{3}+1.239x_{2}x_{3}-1.122x_{1}x_{2}\left(x_{1}-x_{2}\right)\;-0.838x_{1}x_{3}\left(x_{1}-x_{3}\right)+1.243x_{2}x_{3}\left(x_{2}-x_{3}\right)-13.236x_{1}x_{2}x_{3}$$

#### 3.2.4. Influence of the Combined PFA and SF on the Non-Evaporable Water Content

**Figure 12:**
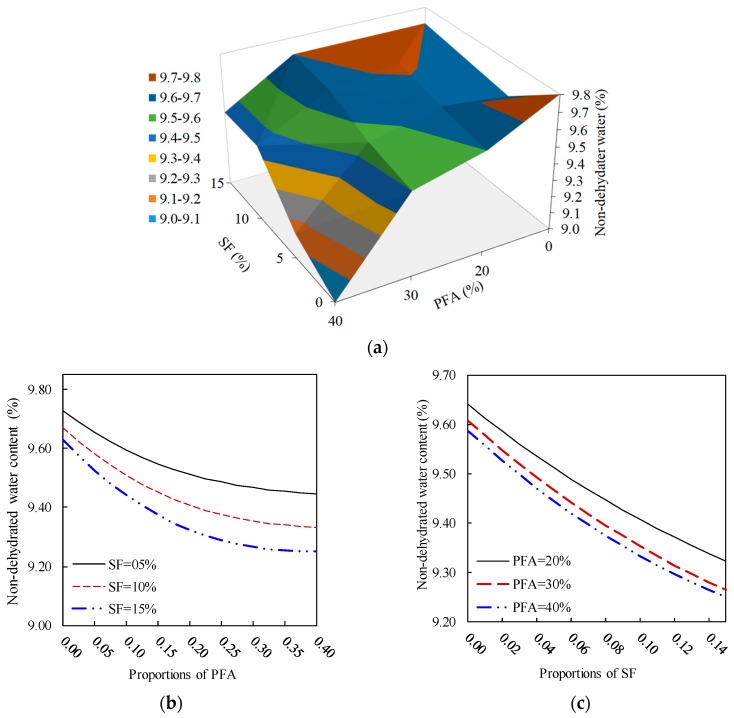
Effect of the PFA and SF on the 28-day non-dehydrated water content of ternary blended systems: (**a**) response surface, (**b**) constant SF, (**c**) constant PFA.

Figure 12 shows the combined impact of blending PFA and SF with PC on the non- evaporable water content of the paste. Figure 12a exhibits the PFA and SF dependent response surface of the non-evaporable water content that was experimentally observed. Unlike the CH content, Figure 12b reveals that for a constant level of SF, as the PFA amount increase the non- evaporable water content decreases. This finding enhances the reliability of the prediction model as comparable experimental observation was recorded (Figure 12a). Additionally, Figure 12b exhibits that the reduction percentages of the non-dehydrated water content for 5%, 10% and 15% were 2.76%, 3.40% and 3.90%, respectively. Therefore, the influence of SF on the non- evaporable water content becomes noticeable as its content increases. Moreover, Figure 12c shows that the effect of SF and PFA on the non-evaporable water content is similar to that for the CH content of ternary blended pastes. For a constant dose of PFA, this effect is represented by the reduction in the non-evaporable water content as the SF replacement level increases, having a descending trend-line.

From Figure 12b, the effect of PFA content on the presence of non-evaporable water decreases with an increase in PFA content. As PFA content increases, the variation in its presence is significant. The presence of SF content also demonstrates reduction in non-evaporable water content with an increase in SF content; however, it tends to diverge with presence of PFA content. For example, at 5% SF + 0% PFA and 15% SF + 0% PFA the non-evaporable water contents are about 9.6% and 9.2%, respectively whereas by adding 40% PFA to these mixes the non-evaporable water contents reported is about 9.5% and 9.3%, respectively. Although, this change is not significant. Similar trends are shown in Figure 12c, which shows insignificant effect of change of PFA from 20% to 40%. However, inclusion of SF is showing significant change from 5% to 15%. Clearly, inclusion of both SF and PFA reduced non-evaporable water content, however, it is limited to the proportion of SF and PFA and to the optimum range of SF and PFA, which is approximately similar to that of the 28-day CH content (Figure 12b,c) within the range of 8% to 10% and 25% to 30%, respectively.

### 3.3. Microstructural Analysis of Binary and Ternary Mixtures

**Figure 13:**
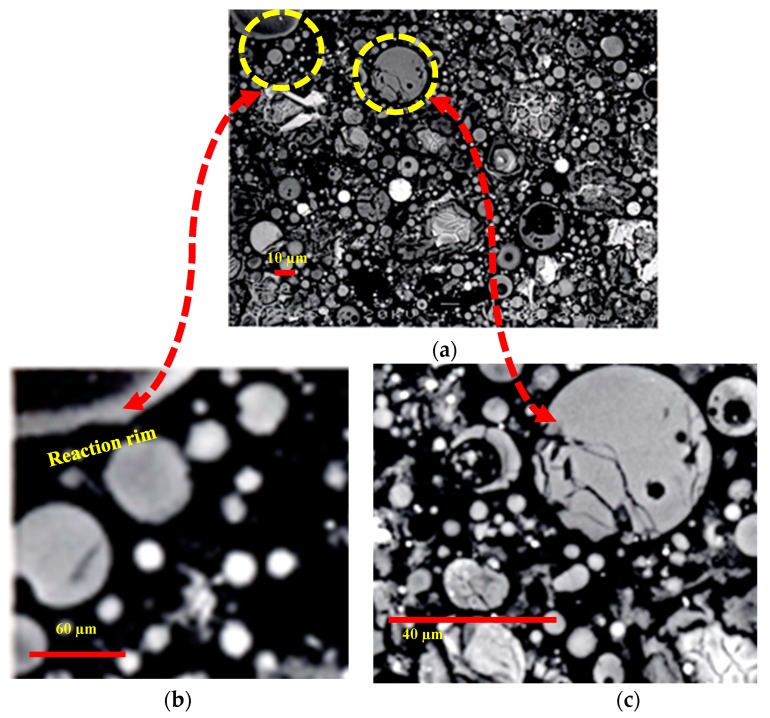
Binary paste mixture containing; (**a**) 40% PFA, (**b**) 6× enlarged spots reaction rims around PFa, (**c**) 4× enlarged PFa particle cracked due to pozzolanic reaction.

**Figure 14:**
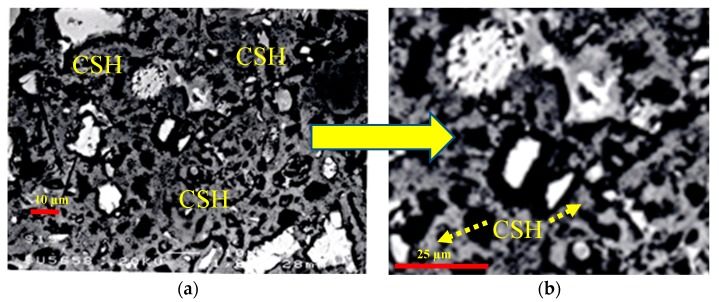
BSE of PC-SF binary mixture containing (**a**) 15% SF, (**b**) 2.5× enlarged spot showing CSH.

**Figure 15:**
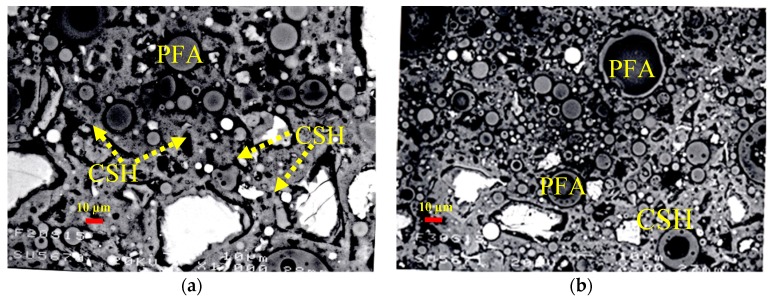

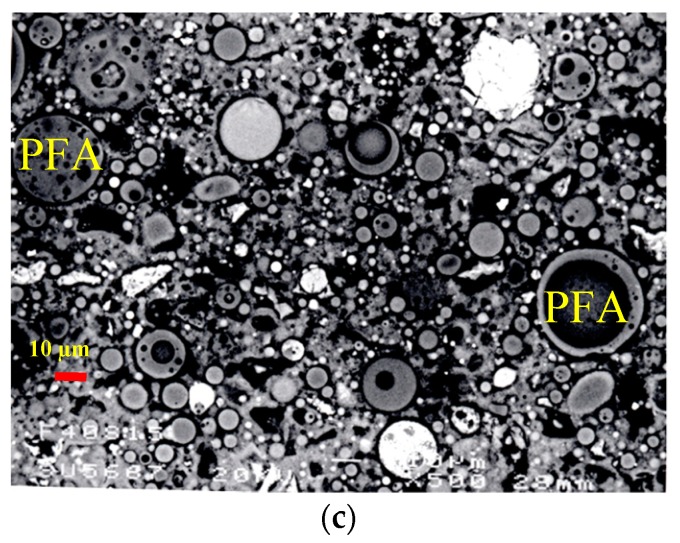
Microstructural analysis of ternary mixtures of PC with PFA and SF; (**a**) 20% PFA + 15% SF, (**b**) 30% PFA + 15% SF, (**c**) 40% PFA + 15% SF.

The control, binary and ternary mixtures were investigated for microstructure using EBSD technique. The presence of cenospheres particles in PFA increase with increasing PFA content from 20% to 40%. A typical example of the highest content of the binary paste mixture with 40% PFA is shown in Figure 13a. The formation of reaction rim around PFA cenospheres becomes obvious at this curing age of hydration, as illustrated in in Figure 13b. The disintegration of PFA particle has also been noticed, as confirmed in Figure 13c. The pozzolanic reaction has been clearly initiated, which can be further proceeded. Similarly, the incorporation of SF from 5% to 15% into PC accelerates the hydration process. The effect of 15% SF on the formation of additional CSH surrounding PC particles is obviously presented in Figure 14a and clarified through magnification in Figure 14b. The microstructural analysis of ternary mixtures of PC, PFA and SF has revealed that the presence of SF becomes effective in the formation of additional CSH when SF exceeds 5% while the reaction of PFA particles become difficult at elevated PFA level of 40%. Typical examples for the hydration reaction of ternary mixtures of 20% PFA + 15% SF (F20S15), 30% PFA + 15% SF (F30S15) and 40% PFA and 15% SF (F40S15) are given in Figure 15. The additional formation of CSH becomes obvious at lower PFA of 20% and it fades out with higher PFA levels of 30% and 40% due to concomitant lowering PC content.

## 4. Conclusions

In the current research, the hydration parameters of ternary blended pastes incorporating PFA and SF were investigated by TGA, microstructural analysis and simplex-lattice modelling techniques. The conclusions drawn from the above two approaches are summarized as follows:

1. At early age (up to seven days), the consumption of CH is dominant due to incorporation of SF with PC in a paste, however, after that insignificant increase was observed. The introduction of SF markedly decreases the amount of CH once after that by few hours (up to one-day) of initiation of the hydration reaction. Up to seven-days, the pastes with SF displayed high non- evaporable water content with respect to the reference paste. This increase is associated with the production of extra CSH gel as a result of the accelerated pozzolanic reaction between SF and CH.
2. The quadratic and cubic simplex-lattice design models for the prediction of CH content and non-evaporable water content exhibited well performance as most of the predicted results were close to the observed results at 28 days. Based on these models, it was remarked that the influence of PC on the CH content is notably more than those of PFA and SF, however, the CH content of the paste containing only PFA is comparable to that with merely SF. However, the two pozzolanic materials have comparable influence on the non-evaporable water content. Moreover, the ternary blend of PC-PFA-SF has the “synergistic” consequence on the release of CH of the paste. Conversely, this ternary blend has the “antagonistic” effect on the discharge of non-dehydrated water of the paste.
3. Microstructural analysis revealed the accelerated hydration due to the presence of SF and the formation of additional CSH around PC grains. The hydration reaction of PFA takes place on the surface of its cenospheres initiated by surface dissolution and formation of additional CSH. The efficiency of SF compared to PFA was highly notable. SEM technique is an important supportive tool to visualize the microstructure approach of multi-cementitious systems’ hydration and modelling.

## Figures and Tables

**Table 1:** Proportions of Portland cement (PC), pulverized fuel ash (PFA) and silica fume (SF) of the developed mixtures.

No. of Mix.	Designation	PC	PFA	SF	SP (%, D.E.)
1	CONTROL	1.00	0.00	0.00	0.41
2	F20	0.80	0.20	0.00	0.34
3	F30	0.70	0.30	0.00	0.29
4	F40	0.60	0.40	0.00	0.25
5	S5	0.95	0.00	0.05	0.41
6	S10	0.90	0.00	0.10	0.43
7	S15	0.85	0.00	0.15	0.48
8	F20S5	0.75	0.20	0.05	0.34
9	F30S5	0.65	0.30	0.05	0.32
10	F40S5	0.55	0.40	0.05	0.31
11	F20S10	0.70	0.20	0.10	0.40
12	F30S10	0.60	0.30	0.10	0.38
13	F40S10	0.50	0.40	0.10	0.35
14	F20S15	0.65	0.20	0.15	0.42
15	F30S15	0.55	0.30	0.15	0.39
16	F40S15	0.45	0.40	0.15	0.36

**Table 2:** Calcium hydroxide content (percentage) of binary and ternary cement pastes at various ages.

Designation	1-Day	3-Day	7-Day	28-Day	90-Day	180-Day
CONTROL	12.32	13.96	14.61	16.17	16.44	16.80
F20	11.95	13.24	12.81	12.71	12.66	12.40
F30	10.68	12.46	11.42	10.99	10.77	10.26
F40	9.52	10.88	8.91	8.16	7.45	5.91
S5	10.32	11.37	11.57	12.01	12.19	11.74
S10	8.87	9.67	10.28	10.04	9.71	9.22
S15	7.77	8.54	7.80	7.35	6.67	5.12
F20S5	10.86	11.51	10.95	10.36	10.03	9.28
F30S5	10.25	11.23	9.45	8.82	7.89	7.27
F40S5	8.85	9.88	8.70	7.91	7.35	6.43
F20S10	7.22	8.80	8.95	8.52	8.25	8.00
F30S10	6.44	7.51	7.78	7.72	7.77	7.82
F40S10	5.61	6.38	6.46	6.76	6.92	7.28
F20S15	5.62	6.30	6.93	6.68	6.67	6.66
F30S15	5.44	5.86	6.06	6.26	6.46	6.05
F40S15	4.16	4.65	5.45	5.14	4.83	4.83

**Table 3:** Non-dehydrated water content (percentage) of binary and ternary cement pastes at various ages.

Designation	1-Day	3-Day	7-Day	28-Day	90-Day	180-Day
CONTROL	4.73	6.90	7.97	9.80	10.10	10.10
F20	4.30	6.50	7.95	9.60	9.85	9.90
F30	4.00	6.20	7.93	9.50	9.75	9.80
F40	3.75	5.90	7.70	9.00	9.30	9.40
S5	4.85	7.13	8.50	9.60	9.84	9.80
S10	5.07	7.50	8.67	9.65	9.85	9.85
S15	5.19	7.65	8.75	9.70	9.90	9.90
F20S5	4.50	6.83	8.00	9.65	9.90	9.90
F30S5	4.30	6.70	8.10	9.56	9.81	9.82
F40S5	4.00	6.62	8.10	9.15	9.41	9.41
F20S10	4.60	7.32	8.55	9.72	9.92	9.92
F30S10	4.50	7.10	8.45	9.67	9.87	9.87
F40S10	4.40	7.10	8.12	9.45	9.72	9.72
F20S15	4.70	7.46	8.70	9.70	9.92	9.92
F30S15	4.56	7.30	8.56	9.70	9.90	9.90
F40S15	4.45	7.19	8.11	9.45	9.72	9.72

**Table 4:** Measured and predicted CH and non-dehydrated water contents of the developed mixtures.

Mix.	Components (Coordinates)						Response									
	Actual			Pseudo ^(*)^			CH (%)					Non-Dehydrated Water (%)				
	$z_{1}$	$z_{2}$	$z_{2}$	$x_{1}$	$x_{2}$	$x_{3}$	Obs. ^(**)^	Predicted (Pred.)				Obs. ^(**)^	Predicted			
								$f_{CH}^{\left(2\right)}$	Pred./Obs.	$f_{CH}^{\left(3\right)}$	Pred./Obs.		$f_{W}^{\left(2\right)}$	Pred./Obs.	$f_{W}^{\left(3\right)}$	Pred./Obs.
Control	1.00	0.00	0.00	1	0	0	16.17	16.17	1.00	16.17	1.00	9.80	9.80	1.00	9.80	1.00
F20	0.80	0.20	0.00	2/3 (1/2)	1/3 (1/2)	0	12.71	17.45	1.37	14.29	1.12	9.60	9.71	1.01	9.60	1.00
F30	0.70	0.30	0.00	1/3	2/3	0	10.99	14.78	1.34	11.00	1.00	9.50	9.44	0.99	9.50	1.00
F40	0.60	0.40	0.00	0	1	0	8.16	8.16	1.00	8.16	1.00	9.00	9.00	1.00	9.00	1.00
S5	0.95	0.00	0.05	0	2/3 (1/2)	1/3 (1/2)	12.01	11.67	0.97	10.04	0.84	9.60	9.46	0.99	9.60	1.00
S10	0.90	0.00	0.10	0	1/3	2/3	10.04	11.40	1.14	12.01	1.20	9.65	9.69	1.00	9.65	1.00
S15	0.85	0.00	0.15	0	0	1	7.35	7.35	1.00	7.35	1.00	9.70	9.70	1.00	9.70	1.00
F20S5	0.75	0.20	0.05	1/3	0	2/3	10.36	-(***)	-	9.82	0.95	9.65	9.56	0.99	9.65	1.00
F30S5	0.65	0.30	0.05	2/3 (1/2)	0	1/3 (1/2)	8.82	-(***)	-	8.55	0.97	9.56	9.60	1.00	9.56	1.00
F40S5	0.55	0.40	0.05	1/3	1/3	1/3	7.91	5.28	0.67	11.93	1.51	9.15	9.62	1.05	9.15	1.00
F20S10	0.70	0.20	0.10	7/20	1/2	3/20	8.52	10.91	1.28	8.16	0.96	9.72	9.56	0.98	9.80	1.01
F30S10	0.60	0.30	0.10	3/10	7/20	7/20	7.72	5.59	0.72	7.35	0.95	9.67	9.61	0.99	9.60	0.99
F40S10	0.50	0.40	0.10	3/10	1/3	27/73	6.76	4.96	0.73	8.55	1.26	9.45	9.62	1.02	9.50	1.01
F20S15	0.65	0.20	0.15	9/20	3/10	1/4	6.68	5.98	0.90	8.16	1.22	9.70	9.63	0.99	9.00	0.93
F30S15	0.55	0.30	0.15	3/10	3/10	2/5	6.26	4.00	0.64	7.35	1.17	9.70	9.62	0.99	9.60	0.99
F40S15	0.45	0.40	0.15	1/5	2/5	2/5	5.14	7.00	1.36	8.55	1.66	9.45	9.60	1.02	9.65	1.02
Minimum							4.00	4.00	0.64	7.35	0.84	9.00	9.00	0.98	9.00	0.93
Average							9.10	9.34	1.01	9.84	1.11	9.56	9.58	1.00	9.52	1.00
Maximum							16.17	17.45	1.37	16.17	1.66	9.80	9.80	1.05	9.80	1.02
Range							11.03	13.45	0.73	8.82	0.82	0.80	0.80	0.07	0.80	0.09

* The pseudo-components for the quadratic model are shown in brackets; ** Observed result; *** Negative value (not acceptable).
